# Effects of Thermal Regimes, Starvation and Age on Heat Tolerance of the Parthenium Beetle *Zygogramma bicolorata* (Coleoptera: Chrysomelidae) following Dynamic and Static Protocols

**DOI:** 10.1371/journal.pone.0169371

**Published:** 2017-01-04

**Authors:** Frank Chidawanyika, Casper Nyamukondiwa, Lorraine Strathie, Klaus Fischer

**Affiliations:** 1 Agricultural Research Council, Plant Protection Research Institute, Weeds Division, Hilton, South Africa; 2 Department of Biology and Biotechnological Sciences, College of Science, Botswana International University of Science and Technology (BIUST), Palapye, Botswana; 3 Zoological Institute & Museum, University of Greifswald, Greifswald, Germany; USDA Agricultural Research Service, UNITED STATES

## Abstract

Temperature and resource availability are key elements known to limit the occurrence and survival of arthropods in the wild. In the current era of climate change, critical thermal limits and the factors affecting these may be of particular importance. We therefore investigated the critical thermal maxima (CTmax) of adult *Zygogramma bicolorata* beetles, a biological control agent for the invasive plant *Parthenium hysterophorus*, in relation to thermal acclimation, hardening, age, and food availability using static (constant) and dynamic (ramping) protocols. Increasing temperatures and exposure times reduced heat survival. In general, older age and lack of food reduced heat tolerance, suggesting an important impact of resource availability. Acclimation at constant temperatures did not affect CTmax, while fluctuating thermal conditions resulted in a substantial increase. Hardening at 33°C and 35°C improved heat survival in fed young and mid-aged but only partly in old beetles, while CTmax remained unaffected by hardening throughout. These findings stress the importance of methodology when assessing heat tolerance. Temperature data recorded in the field revealed that upper thermal limits are at least occasionally reached in nature. Our results therefore suggest that the occurrence of heat waves may influence the performance and survival of *Z*. *bicolorata*, potentially impacting on its field establishment and effectiveness as a biological control agent.

## Introduction

In ectotherms, the capacity for thermal regulation is limited such that ambient temperature has direct consequences on overall fitness [1]. This is because variation in temperature directly influences physiological processes in insects such as metabolic rates [1,2]. Consequently, recent climate change has caused range shifts [3,4] and phenological changes in many insects [5–7].

Facing the pervasive impact of temperature on ectotherm fitness, it is not surprising that organisms may rapidly adjust to thermal variation through mostly reversible physiological mechanisms (reviewed in Teets & Denlinger, 2013) [8]. For example, short-term ‘hardening’, involving a pre-exposure to sublethal high (heat shock) or low (cold shock) temperatures, have been shown to improve survival under subsequent more extreme temperatures [9–11]. Similar plastic responses can also be induced by longer pre-exposure times prior to exposure in extreme temperatures, referred to as acclimation [12–15].

Acclimatory responses can be induced within hours, days or even longer periods, depending on species [15–19]. Improved stress tolerance following thermal acclimation is typically considered to represent adaptive phenotypic plasticity, often referred to as beneficial acclimation hypothesis (BAH). Accordingly, acclimation of an organism to a particular environment should confer fitness advantages upon exposure to similar environmental conditions as compared with individuals acclimated to different conditions [12–20]. Interestingly, a number of studies have also shown cross-tolerance in insects. such that a pre-exposure to heat may also improve cold tolerance or *vice versa* [9,21,22]. Furthermore, exposure to stressors other than temperature such as starvation, desiccation and hypoxia may also affect thermal tolerance [23–27]. For example, starvation reduced cold tolerance in *Tribolium castaneum* (Coleoptera: Tenebrionidae) whilst heat tolerance remained unaffected [27]. In contrast, feeding in locusts *Locusta migratoria* (Orthoptera: Acrididae) resulted in poor cold tolerance [28].

The fitness advantages conferred by temperature-induced plasticity depends on a host of factors (reviewed by Sgrò *et al*., 2016) [29], including the type, magnitude, timing, and duration of stress [30–34]. Some studies have shown that thermal plasticity is also influenced by age [32,33] and ontogeny [21,35], though our knowledge on such effects are still poor especially due to partly contrasting results [35,36]. For example, while aging resulted in improved cold tolerance in the wingless fly *Anatalanta aptera* (Diptera: Sphaeroceridae) [37], it decreased cold tolerance in the red flour beetle *T*. *castaneum* (Coleoptera: Tenebrionidae) [33]. In the fruit flies *Ceratitis capitata* and *C*. *rosa*, (Diptera: Terphritidae) aged flies had both poor heat and cold tolerance [32]. In the case of ontogenetic variation, it is generally thought that less mobile life-stages are more physiologically plastic as they cannot easily cope with extreme environments through behavioural thermoregulation [38]. However, physiological mechanisms have been recently reported to be more important in mitigating the damage caused by heat than behavioural responses in several insects [39–41].

Because of the non-linearity of thermal performance curves [42] and potentially differential physiological responses and survival rates elicited by constant and fluctuating temperatures among arthropods, there has been a substantial debate on the validity and ecological relevance of the methods used to determine thermal tolerance under laboratory conditions [43–48]. Investigating plastic responses and survival under constant temperatures is generally regarded as being less ecologically relevant [44,48] despite the fact that several studies showed physiological responses that enable survival under extreme thermal conditions following exposure to constant temperatures [49–51]. For assessing heat tolerance, assays involving a gradual temperature change, referred to as dynamic (ramping) protocols, have been widely regarded as being more ecologically relevant, as they better mimic typical field conditions [17,47,48]. However, they have also been criticized for not accounting for potentially confounding effects of starvation or dehydration during the assay [46].

From an applied biological control perspective, the thermal performance of mass-reared insects facing novel environments upon release in the wild has long been a source of disquietude [15,52–55]. Several studies have argued that mass-reared insects, typically kept under constant optimal environments, may struggle under field thermal conditions [15,17,53]. It is therefore especially important to understand the physiological responses to thermal variation in insects used in biological control, as it may help to optimize rearing and release protocols in order to enhance field performance [55].

Against this background, we here use the herbivorous beetle *Zygogramma bicolorata* Pallister (Coleoptera: Chrysomelidae), which is used as a biocontrol agent of the invasive neotropical weed, *Parthenium hysterophorus* L. (Asteracea: tribe Heliantheae, subtribe Ambrosinae). The beetle is native to Mexico and, following assessment of its suitability, was introduced into South Africa in 2013 for the biological control of *P*. *hysterophorus*. Despite initial evidence of *Z*. *bicolorata* being an efficient defoliator soon after release, its field establishment in South Africa remains capricious and overall limited (ARC-PPRI *unpublished data*). We therefore suspected that environmental stress and thermal variability influence survival and field performance of *Z*. *bicolorata*. The aim of this study was thus to determine upper thermal limits and the influence of age, feeding status, and thermal history on heat tolerance in adult *Z*. *bicolorata* using both dynamic (inability to move after gradual temperature increase exposure) and static (survival after constant heat exposure) protocols. Including food availability seems important, as in nature animals often experience thermal stress in concert with other environmental stressors such as host plant quality and availability [1,56]. Specifically, we set out to test the following hypotheses: (1) Heat tolerance shows plastic responses to acclimation and hardening, with beetles benefitting from pre-exposure to warmer temperatures; (2) Heat tolerance decreases with age due to senescence and resource depletion; (3) Food deprivation reduces heat tolerance due to the energetic costs associated with stress responses; (4) Static compared with dynamic heat exposure, results in poorer heat tolerance due to limited capacity for acquiring plasticity that is attained during gradual dynamic temperature change.

## Materials and Methods

### Ethics statement

This study did not involve any endangered or protected species. Initial stock culture of the beetles was obtained with permission and cooperation from the Queensland Department of Primary Industries and Fisheries, Australia. The beetles were then first introduced into South Africa for risk assessments under quarantine on permit no. P0015341 from the Department of Agriculture Forestry and Fisheries, South Africa. The use of Makhathini Research Station where field temperature data was recorded was permitted by the KwaZulu-Natal Department of Agriculture and Rural Department, South Africa as part of a long term study monitoring the impact of introduced exotic insects on *P*. *hysterophorus*.

### Study organism

*Zygogramma bicolorata* beetles originally from Mexico were collected from well-established field populations in Central Queensland, Australia (24° 31’S, 148° 34’E) in 2005, and introduced into quarantine in South Africa to start a laboratory culture. Since then, a culture has been maintained at the Agricultural Research Council’s Plant Protection Research Institute (ARC-PPRI), Cedara Campus, KwaZulu-Natal province. Beetles were reared under semi-field conditions in a glasshouse following the procedures outlined in McConnachie (2015) [57]. In brief, beetles were maintained on *P*. *hysterophorus* plants in steel-framed cages (50 x 50 x 80 cm) covered with thrips screening gauze. Environmental conditions during rearing ranged between 20 and 29°C and 60 to 100% relative humidity, with all beetles used in the experiments below being reared under the same conditions. Host plants were propagated in plastic pots (18 x 10 cm) under drip fertigation for 4–5 weeks before being introduced into the rearing cages. The beetles were allowed to oviposit on the plants, and emerging larvae were separated from adults and allowed to develop through their 4 instar stages in larval rearing cages until pupation. Development time from hatching until adult eclosion is approximately 28 days. Adult beetles were reared in high numbers (ca. 100 individuals per cage, with up to 2000 in culture simultaneously) to avoid inbreeding. Defoliated plants were replaced with fresh ones as necessary. To investigate plasticity in thermal tolerance, five experiments were carried out as outlined below.

### Experiment 1: Age differences in heat survival under constant temperatures

Upper lethal temperatures were determined by assessing survival after exposure to a series of constant temperature and time combinations using programmable water baths (Haake Phoenix II, Haake C25P) as previously described ([9] and Table 4 in [22]). Three adult age groups (3–4, 12–14 and 34–36 days old) were assayed to determine the impact of age on survival by independently exposing individuals for 1, 2 or 4 hrs to 10 different temperatures (34, 36, 38, 40, 42, 44, 46, 48, 50, 52°C) to determine mortality (ranging between 0 to 100%). Adult *Z*. *bicolorata* beetles can live for up to 2 years with diapausing periods of up to 6 months in winter and autumn [58]. However, under benign laboratory conditions, without any diapause induction, the beetles die within 3 months. Here, the 3 age-groups were chosen as they fall within the range that is typically used for field releases. For each temperature by time treatment and age group, 10 beetles, replicated 5 times (N = 50), were placed in 60 ml propylene vials closed with perforated lids. Moistened strips of filter paper were placed into each vial to maintain relative humidity (RH) at above 80% to avoid desiccation. After heat exposure, beetles were retrieved from the water baths and immediately transferred to a Labcon (model: FSIM-RH20; accuracy: ± 0.2°C; Labcon Laboratory Equipment, South Africa) growth chamber set at 26±1°C for 12 hrs to allow for recovery. *Parthenium hysterophorus* leaf cuttings were added to the vials to allow surviving beetles to feed during this period. Survival was scored after 12 hrs as the ability to resume feeding and/or to show a locomotory response to stimuli such as mild prodding with a soft brush. All beetles were discarded after the assays.

### Experiment 2: Effects of hardening, age, and feeding on heat survival under static heat exposure

We assayed hardening responses using exposure to either 19, 33 or 35°C for 2 hrs (see e.g. Chidawanyika & Terblanche, 2011) [9] across beetles aged 3–4, 12–14 and 34–36 days old and in relation to feeding treatment. The high hardening temperatures were chosen as in most insect taxa hardening responses can be induced after a pre-exposure to temperatures 5–10°C below the discriminating temperature (44°C here), whilst the lower temperature was included to test for cross resistance [9,22]. Prior to hardening, beetles were randomly divided into two feeding treatments, having either access to their host-plant and water *ad libitum* or to water only for 3 days prior to assays. In all cases, water for beetles was provided as a moistened wad of paper towel. Following hardening, heat tolerance was tested using exposure to 44°C for 3 hrs (equaling ~ 25% survival based on the results of experiment 1). Control beetles were held at 25°C throughout. Survival was scored as in experiment 1.

### Experiment 3: Effects of thermal acclimation and hardening on CTmax

**Fig 1:**
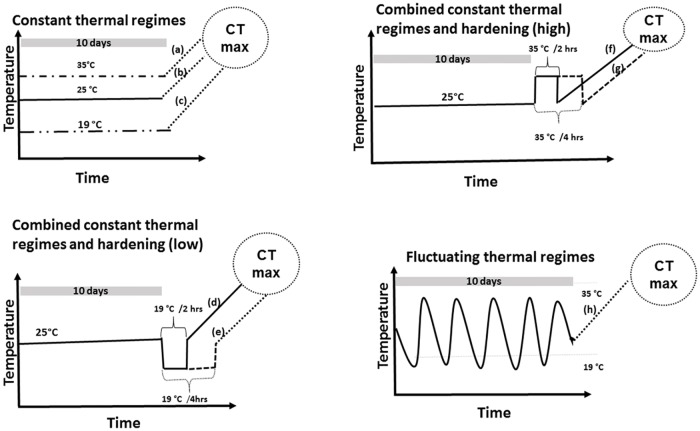
Schematic diagram of protocols for acclimation treatments. Eight different thermal acclimation treatments were used in experiment 3 before measuring the critical thermal maxima (CTmax) for *Zygogramma bicolorata*. Three treatments involved acclimation at constant temperatures of 35°C (a), 25°C (b) and 19°C (c) for 10 days. Four other treatments combined acclimation at 25°C for 10 days with hardening. The respective beetles were hardened at 19°C for 2 (d) or 4 (e) hours or at 37°C for 2 (f) or 4 (g) hours. In the last treatment, beetles were acclimated using daily fluctuating temperatures between 19 and 35°C.

To test for the impact of thermal history on CTmax, freshly eclosed beetles were randomly allocated to 8 thermal treatments for 10 days before assaying CTmax (cf. Fig 1), using Labcon programmable growth chambers (model: FSIM-RH20; accuracy: ± 0.2°C; Labcon Laboratory Equipment, South Africa; cf. Chidawanyika & Terblanche (2011) [9]. In brief, three treatments were acclimated at constant temperatures of either 19, 25 (control) or 35°C. Beetles of treatments 4 to 7 were all kept at 25°C until day 10. On day 10, beetles were divided into four hardening treatments, being afterwards exposed to 19 or 35°C for 2 or 4 hrs. In the last treatment, individuals were exposed to fluctuating temperatures ranging between 19 and 35°C within a diurnal cycle for 10 days. During the 10 days of acclimation, humidity was maintained at 60% throughout and all beetles had access to water and fresh *P*. *hysterophorus* host material for feeding *ad libitum*. To score CTmax, beetles were individually placed into a series of double-jacketed chambers connected to the water bath. After an acclimation period of 15 minutes at 25°C, temperatures started to increase at a ramping rate of 0.25°C min^-1^. A type T copper-constantan thermocouple connected to a digital thermometer (Fluke Cooperation, Australia Ltd., Sydney) was used to verify chamber temperatures. CTmax was scored at the temperature at which beetles were unable to move or self-right after mild probing with a soft brush. At least 30 individuals were assayed for each temperature treatment. Preliminary assays had shown no sex-induced differences in CTmax [59], hence a mixture of males and females (50:50) was used for all the assays.

### Experiment 4: Effects of age and feeding on CTmax

To assess the impact of age and feeding status on the CTmax, 3 age (3–4, 12–14 and 34–36 day old beetles) and 2 feeding (starved or fed) treatments were used. Whilst beetles for the starved treatment were denied access to their host plant 3 days before the onset of experiments, fed beetles had access throughout. CTmax was determined for at least 30 beetles per treatment using the method described for experiment 3.

### Experiment 5: Field temperature measurements

Temperature data were recorded at the KwaZulu-Natal Department of Agriculture and Rural Development’s Makhathini research station (27° 26' 35.8" S; 32° 09' 41.5"E; altitude: 92m above sea level), in northern KwaZulu-Natal province using Maxim Integrated _™_ DS1923 Hygrochron Temperature/Humidity Logger iButtons (-20°C to +85°C, Acc 0.5°C) (via Fairbridge Technologies, South Africa) between November 2015 and February 2016, during the peak growth season for *P*. *hysterophorus*. The iButtons were set within wild populations of *P*. *hysterophorus*, the target plants of the beetles, at 1m above ground (canopy) and at ground level where they were covered with leaf litter. In both cases, sampling frequency was set at 30 minutes throughout the entire recording period.

### Statistical analyses

Due to non-normality of errors and non-homogeneity of variances in the data from experiment 1, the impact of temperature, exposure time and age on the survival of beetles was analysed using generalized linear models (GLM) in R 3.2.3 [60], assuming a binomial distribution (dead or alive) with a probit link function and a single correction for overdispersion [61,62]. Similarly, effects of hardening, age and feeding treatment (experiment 2) were analyzed with a GLM with a quasibinomial distribution of errors and a logit link function (e.g. Chidawanyika & Terblanche, 2011) [8]. One-way ANOVAs were used to assess the impact of thermal history on CTmax in experiment 3 [63]. The impact of age and feeding status in experiment 4 was assessed using two-way ANOVAs. In the latter cases, key assumptions for ANOVAs were met. Normality of the data was tested using the Shapiro-Wilk test whilst equality of variances was confirmed using the Hartley-Bartlett test.

## Results

### Experiment 1: Age differences in heat survival under constant temperatures

**Fig 2:**
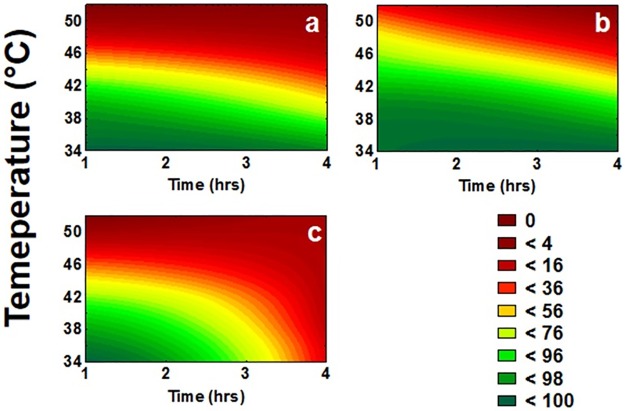
Surface plots for the impact of temperature and exposure time on the survival of *Z*. *bicolorata*. Three age treatments for the beetles were used: (a) 2–3, (b) 14–16, or (c) 34–36 days. Graphs were derived from plotting data of survival after exposing beetles to temperatures ranging from 34 to 52°C for one, two or four hours. For each temperature-time combination, 5 replications of 10 beetles each were used (N = 50). Survival proportions are coded with colour shades where deep red represents 0% survival and dark green represents 100% survival.

The factors age, exposure time, and exposure temperature significantly affected the survival of beetles (Table 1). Survival decreased with increasing temperature and exposure time, and was highest in mid-age and lowest in old beetles (Fig 2). All interactions between age, temperature, and exposure time were also significant (Table 1). Mid-age beetles were least sensitive to increasing temperatures followed by young and finally old beetles (significant age by temperature interaction). Old beetles were more sensitive to exposure time than both other age groups (significant age by exposure time interaction). Thus, survival decreased most rapidly in old beetles with increasing temperatures and exposure times (significant three-way interaction). Finally, exposure time had a larger impact at lower than at higher temperatures (significant exposure time by temperature interaction).

**Table 1:** Results of a Generalised Linear Model (GLM) with a binomial distribution and a logit link function, corrected for overdispersion, investigating the impact of temperature (34 to 52°C), exposure time (1, 2 or 4 hours), and age (3–4, 12–14 and 34–36 days old) on the survival of *Zygogramma bicolorata* adult beetles. DF denotes degrees of freedom; SE = standard error.

Effect	df	Estimate	SE	*Z*	*p*-value
Intercept	1	23.55	7.72	3.07	**<0.001**
Exposure time	2	0.16	0.01	12.19	**<0.001**
Age	2	0.01	0	5.15	**<0.001**
Temperature	9	0.09	0	23.1	**<0.001**
Age x Temperature	12	0.04	0.03	4.25	**<0.001**
Age x Exposure time	5	1.19	0.45	1.27	**0.04**
Exposure time x Temperature	12	1.32	0.38	6.57	**<0.001**
Age x Exposure time x Temperature	15	4.26	1.43	10.1	**<0.001**

### Experiment 2: Effects of hardening, age, and feeding on heat survival under static heat exposure

**Fig 3:**
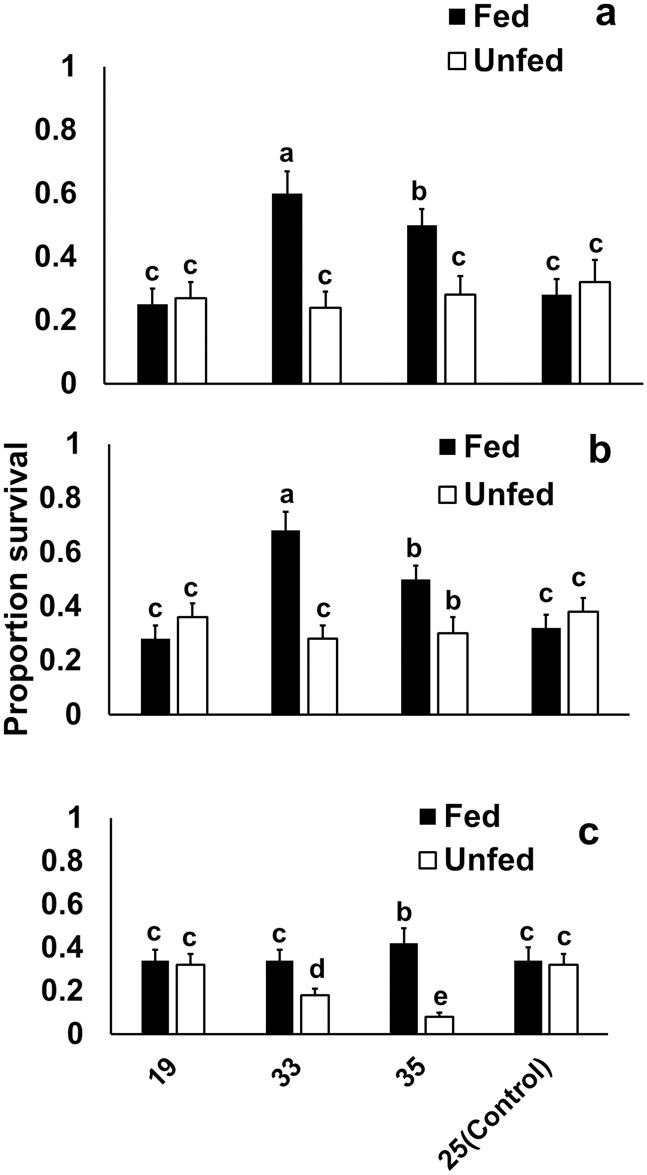
Effect of hardening pre-treatments on survival of starved and fed beetles. Heat (44°C for three hours) survival of fed and starved *Zygogramma bicolorata* aged (a) 2–3, (b) 14–16 or (c) 34–38 days, following hardening at 19, 33 and 35°C for two hours, and a control group held at 25°C. Error bars denote standard error for each data point for a mean of N = 50. Letters above error bars denote statistical significance among different groups.

Hardening temperature and feeding status but not age significantly affected heat survival (Table 2). In general, hardening at 33 or 35°C increased heat survival, and fed beetles showed a higher survival than food-deprived ones (Fig 3). However, positive effects of hardening were restricted to fed beetles, while unfed beetles could not benefit (significant feeding status by hardening temperature interaction). Effects of feeding regime were most pronounced in mid-age beetles (significant age by feeding status interaction). Hardening at 33 and 35°C for two hrs significantly improved heat survival of fed beetles aged 2–3 and 14–16 days, while in the fed beetles aged 34–36 days only hardening at 35°C resulted in improved survival. When being food-deprived, hardening at 33 and 35°C did not change heat survival significantly in the youngest and mid-age beetles, while survival even decreased in old beetles (significant three-way interaction). Throughout, hardening at 19°C did not significantly affect survival.

**Table 2:** Results of a Generalised Linear Model (GLM) with quasibinomial error distribution and a logit link function for the effect of hardening temperature (19, 33 or 35°C for 2 hours), age (3–4, 12–14 and 34–36 days old), and feeding status (access to host plant and water, or water only for three days prior to assay) on heat survival of adult *Zygogramma bicolorata*.

Effect	df	Estimate	SE	*T*	*p*-value
Intercept	1	-2.45	0.45	-5.15	**<0.001**
Hardening temperature	2	0.08	0.02	4.99	**<0.001**
Age	2	0.02	0.02	0.74	0.46
Feeding status	1	2.86	0.66	4.31	**<0.001**
Age x Feeding status	4	0.01	0.03	2.26	**0.02**
Age x Hardening temperature	5	0.00	0.00	-1.51	0.13
Feeding status x Hardening temperature	4	-0.12	0.02	-5.14	**<0.001**
Age x Hardening temperature x Feeding status	7	0.00	0.00	3.21	**0.02**

### Experiment 3: Effects of thermal acclimation and hardening on CTmax

**Fig 4:**
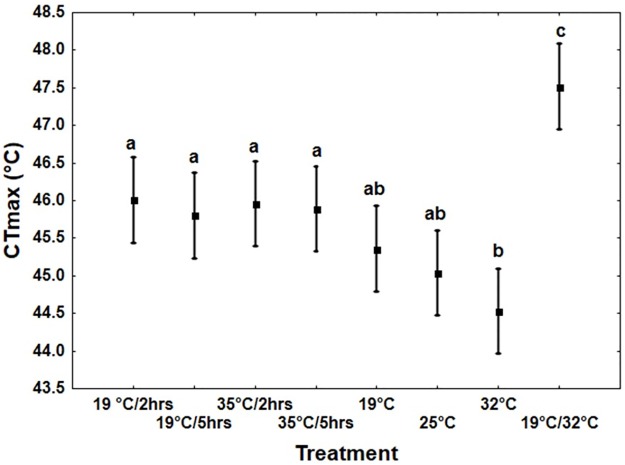
Effects of thermal regime on critical thermal maxima (CTmax) of recently eclosed adult *Zygogramma bicolorata*. Treatments comprised of hardening (**H** treatments) for 2 or 4 hrs, acclimation at constant temperatures (**C** treatments) or under fluctuating day/night thermal cycles (**F** treatment) before assaying CTmax. N = 30 for each treatment, vertical bars denote ±95% CL. Letters above vertical bars indicate significant differences in CTmax.

Thermal regime significantly influenced the CTmax of the beetles (*F*_7, 232_ = 9.3, *p* < 0.0001). Beetles kept under fluctuating temperatures of 19°C/35°C had a higher CTmax compared to all other treatments, while those acclimated to a constant temperature of 35°C showed the lowest value (Fig 4).

### Experiment 4: Effects of age and feeding on CTmax

**Fig 5:**
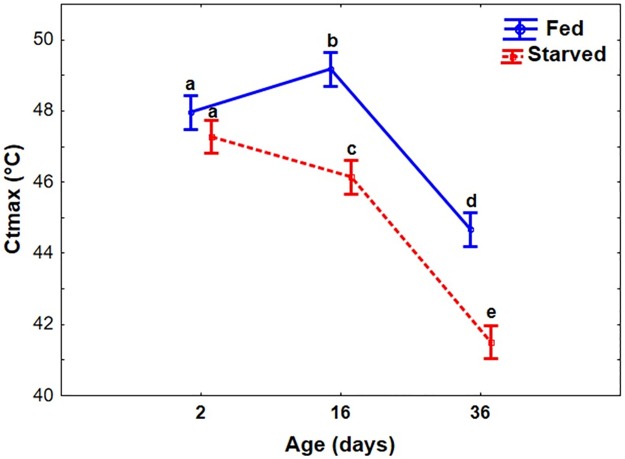
Feeding and starvation effects on the critical thermal maxima (CTmax) of adult *Zygogramma bicolorata* beetles. Assayed beetles were comprised of 3 age treatments (3–4, 12–14 and 34–36 days old). Beetles that were denied access to host plant 3 days prior to assays were regarded as the starved treatment whilst those that had access were considered fed. Data points represent means for *N* = 30 individuals per treatment where vertical bars denote ±95% CL. Letters above data points indicate significant differences in CTmax.

Age (*F*_2,174_ = 241.4, *p* < 0.001) and feeding status (*F*_*1*,174_ = 137.8, *p* < 0.001) significantly affected CTmax, which in general decreased with age and was lower in unfed than in fed beetles (Fig 5). In unfed beetles, however, CTmax consistently decreased with increasing age, while fed beetles reached the highest CTmax in the intermediate age group of 14–16 days (significant age x feeding status interaction; *F*_2,174_ = 17.1, *p* < 0.001).

### Experiment 5: Field temperature measurements

**Fig 6:**
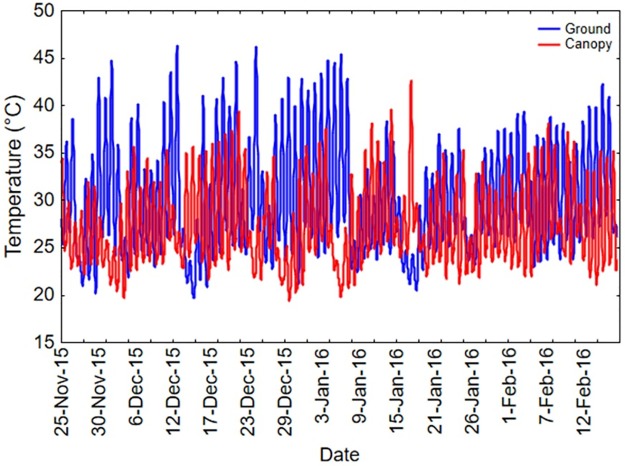
Microclimatic temperature recordings at Makhathini Research Station, KwaZulu-Natal province, South Africa. iButtons (Maxim Integrated_TM_; Model DS 1923, via Fairbridge Technologies, South Africa; 0.5°C precision; 30 min sampling frequency) were used to record data at ground and canopy level (1m above ground) within *Parthenium hysterophorus* stands. Graph was plotted using temporal averages for single day intervals.

Recordings between November 2015 to February 2016 at Makhathini Research Station revealed that temperature ranged from 18.9 to 49.9°C and 16.1 to 47.5°C at ground and *P*. *hysterophorus* canopy levels (1m above ground), respectively (Fig 6). Mean ground and canopy temperature was 30±0.1°C and 27.3±0.1°C, respectively.

## Discussion

The current study confirms that temperature variability has profound effects on survival and physiological performance of Z. *bicolorata*. Exposure to constant temperatures ranging between 34 and 52°C for 1–4 hours revealed that 50% of the beetles died at about 44°C. As expected, mortality increased with increasing temperature and exposure time in keeping with previous studies on other insect species [9,64]. Thus, the heat stress experienced was a function of both absolute temperature and the duration of exposure, suggesting that heat damage is higher at higher temperatures and also accumulates over time [65,66]. At very high temperatures, though, the impact of exposure time becomes less important, as proteins will rapidly denature causing irreversible damage [1,56].

In line with our first hypothesis, we could reveal plastic responses in heat tolerance, especially the capacity for rapid heat hardening, in *Z*. *bicolorata*. Pre-exposure to 35°C generally improved survival, being up to two-times higher compared with controls in fed beetles (for starved beetles see below). Hardening at 33°C elicited similar hardening responses except in old beetles (Fig 3). Such ability for rapid heat hardening has been reported in various studies [9,33,50]. Pre-exposure to low temperatures may also enhance survival at high temperatures, typically referred to as cross-tolerance [9,22]. In the present study, however, there was no improvement in survival after pre-exposure to 19°C, suggesting that cross-tolerance did not occur at this particular thermal exposure [cf. 9,44] (reviewed in King & McRae, 2015) [25]. Perhaps 19°C was not sufficiently low to induce the required stress response, as most studies that have shown cross-tolerance used much lower including sub-zero temperatures in pre-treatments [9]. Here, 19°C was nevertheless used as this is the average temperature prevailing during lengthy transportation of mass-reared beetles in cooler boxes with freezer packs for field releases. Moreover, sub-zero temperatures hardly ever occur in the targeted ecosystem, in which the lowest and most frequent low temperature was 15 and 20°C, respectively, in this part of the invaded range (Fig 6). Hence, sub-zero temperatures may not be of any ecological relevance.

Measuring heat tolerance as CTmax using a dynamic protocol though revealed at least partly divergent results. Here, hardening for two or four hours at 35°C did not improve subsequent heat tolerance measured as inability to move (see further below). Furthermore, acclimation at higher constant temperatures did not improve heat tolerance but tended to reduce it (Fig 4). We assume that this is the consequence of the rather long acclimation period of 10 days, with higher temperatures increasing metabolic rates and thus causing an earlier depletion of energy stores needed to fuel stress responses. Interestingly though, the fluctuating thermal regime resulted in a dramatic increase in heat tolerance, as reported in other species [44]. Thus, keeping the beetles under more natural conditions clearly had positive effects on heat tolerance. This might be a consequence of high temperatures enabling high (feeding) activity during daytime, while the low night-temperatures may reduce metabolic losses, although the mechanisms through which fluctuating temperatures improve thermal tolerance are a matter of debate [44,67–73]. The effects of fluctuating temperatures seem further to be sensitive to the amplitude and the mean of the temperatures [44,68,74,75]. Large amplitudes have been suggested to improve heat tolerance [75]. Since the stock of *Z*. *bicolorata* used for the trials was kept under fluctuating thermal conditions for many generations, it is also possible that transmissible trans-generational effects (see discussions in Sgrò *et al*., 2016) [29] favored performance and plasticity under fluctuating thermal regimes. Thus, these findings may indicate an important role of diel thermal fluctuations for signaling risk of future stressful events in insects [76]. This, however, does not incontrovertibly discredit the use of constant acclimation temperatures which have been shown to improve performance even under field conditions [15,17]. Our findings can alternatively be explained by the different fitness consequences of mild stress under chronic (acclimation) and acute (rapid hardening) thermal exposure. Acute exposure to mild stress can actually offer an opportunity for organisms to improve fitness through rapid transient physiological responses [51,77]. Under prolonged exposure, however, the activation of such physiological responses may become costly [73]. It is therefore possible that the fitness of Z. *bicolorata* in our study was compromised by prolonged exposure without transient phases for recovery.

Our study revealed pronounced differences in the way insects respond to dynamic (measuring CTmax) and static (measuring heat survival) protocols (hypothesis 4) [44,64], as hardening did not result in improved CTmax using ramping assays. However, it should be noted that the target traits being compared also differed, being survival after exposure to a heat spell or the ability to move at increasing temperatures. These findings may suggest that immediate exposure to stressfully high (constant) temperatures potentially overestimates thermal tolerance and other temperature-related fitness parameters [70,76,78]. Alternatively, hardening may only affect heat survival but not proxies thereof. Either way, our results strengthen the need to be cautious when interpreting results on heat tolerance, as they are dependent on methodological context [43,79].

Overall, heat tolerance decreased with increasing age as expected (hypothesis 2), with heat survival and CTmax yielding comparable results. Old beetles were the most sensitive to both absolute temperature and exposure time. While this was generally the case in starved beetles, fed beetles showed a more complicated pattern. In two independent experiments using different protocols, heat tolerance was highest in mid-age beetles. We therefore suggest that age-related variation in heat tolerance may be driven by resource availability in some species. In food-deprived beetles, resources declined continuously with age, while young beetles may initially gain mass and store additional resources before effects of senescence become dominant. These results are in line with others showing a negative relationship between age and thermotolerance in insects [32,33,80,81]. However, an increase in thermotolerance with age has also been reported [37], suggesting that responses may be stress- and species-specific. Differences in the indices used to assess thermal tolerance (e.g. CTmax vs time to heat knockdown, CTmin vs chill coma recovery) may add further complexity.

Interestingly, age did not only negatively affect heat tolerance in general, but also the beetles’ ability to respond plastically to heat, as evidenced by detrimental rather than beneficial effects of hardening. This is in keeping with studies on *Drosophila* where the capacity for heat hardening decreased with age [81]. Such age-dependent plasticity in stress tolerance warrants further investigations using different abiotic stressors, as most studies only focus on the impact of age on basal tolerance [82].

In line with the above notion of the key role of resource availability, starved as compared with fed beetles showed generally a reduced heat tolerance in the present study, except perhaps for very young beetles in which starvation may not have a severe impact (hypothesis 3). Interestingly, hardening did not have positive effects in starved individuals, and even negative effects occurred in old beetles. Thus, reduced resource availability clearly compromised thermal tolerance and its plasticity suggesting that these beetles were not able to upregulate physiological defense mechanisms any more, as also demonstrated in Terphritid species [32]. In addition, a recent study on a Drosphilid species demonstrated that both transient and prolonged stress through temperature depletes energy reserves [83].

The implications of this study for the biological control of *P*. *hysterophorus* using *Z*. *bicolorata* are several-fold. First, the upper lethal limits reported here in both dynamic and constant thermal regimes were occasionally reached in the field, suggesting that heat waves may interfere with the performance, survival and ultimately field establishment of *Z*. *bicolorata* in hotter regions or during extreme heat events. These results are thus in contrast with bioclimatic models having generally predicted the region as being suitable for *Z*. *bicolorata* [59], with the caveat that field data for this study were collected during drought conditions, with above average temperatures and minimal rainfall. Field releases should therefore be accompanied by seasonal monitoring to confirm presence and activity of the beetles, especially after extreme thermal events. If necessary, augmentative releases should be carried out to boost field populations. Second, we found some degree of induced heat tolerance following both hardening and acclimation, suggesting the ability to cope with temperature variation to some extent. This aspect warrants further investigation to improve mass-rearing and transportation protocols. Specifically, the role of thermal effects during ontogeny should be explored in the future to gain a more complete understanding of the species`thermal ecology (e.g. Zhang *et al*., 2015) [84]. For the time being, the use of diel temperature fluctuations can be clearly recommended. Third, increasing age generally resulted in poor heat tolerance, stressing the need to release young animals into the field. Fourth, the poor heat tolerance of starved beetles indicates that limited food availability in the field will result in increased vulnerability to thermal variation. Hence, in all likelihood, density-dependent feedback mechanisms mediated by food availability may also determine survival in the field. Populations of *Z*. *bicolorata* may therefore be compromised by extreme heat events and limited host plant density.

In conclusion, our results highlight within-generation variation in heat tolerance in *Z*. *bicolorata*, being affected by thermal regime, food availability and age. More importantly, the study showed pronounced effects of acclimation to constant versus naturally fluctuating temperatures, stressing the importance of diel thermal fluctuations in determining critical thermal limits. Despite evidence for plasticity in heat tolerance, microclimate data suggest that thermal limits may be at least occasionally exceeded. Plastic responses, or lack thereof, induced by acute stress and ramping protocols in this study, also highlights how methodology may influence the outcomes of measures of temperature tolerance. Future studies should thus consider multiple traits [54], including effects during ontogeny as well as transgenerational effects on acquired tolerance. Overall, our study further highlights the complexity of insect responses to thermal variability, in particular when facing multiple stressors. Future studies may also endeavor to explore how the inclusion of multiple stressors, e.g. host plant availability, may help to improve the predictions of bioclimatic envelope models.
